# Chloramine Disinfection of Levofloxacin and Sulfaphenazole: Unraveling Novel Disinfection Byproducts and Elucidating Formation Mechanisms for an Enhanced Understanding of Water Treatment

**DOI:** 10.3390/molecules29020396

**Published:** 2024-01-13

**Authors:** Zhenkun Sun, Zhenyi Chen, Marie Celine Chung Lan Mow, Xiaowen Liao, Xiaoxuan Wei, Guangcai Ma, Xueyu Wang, Haiying Yu

**Affiliations:** College of Geography and Environmental Sciences, Zhejiang Normal University, Yingbin Avenue 688, Jinhua 321004, China; szk19957926052@163.com (Z.S.); c_line_chung@hotmail.com (M.C.C.L.M.);

**Keywords:** chloramine disinfection, product identification, fluoroquinolone, sulfaphenazole, formation potential, influencing factor

## Abstract

The unrestricted utilization of antibiotics poses a critical challenge to global public health and safety. Levofloxacin (LEV) and sulfaphenazole (SPN), widely employed broad-spectrum antimicrobials, are frequently detected at the terminal stage of water treatment, raising concerns regarding their potential conversion into detrimental disinfection byproducts (DBPs). However, current knowledge is deficient in identifying the potential DBPs and elucidating the precise transformation pathways and influencing factors during the chloramine disinfection process of these two antibiotics. This study conducts a comprehensive analysis of reaction pathways, encompassing piperazine ring opening/oxidation, Cl-substitution, OH-substitution, desulfurization, and S−N bond cleavage, during chloramine disinfection. Twelve new DBPs were identified in this study, exhibiting stability and persistence even after 24 h of disinfection. Additionally, an examination of DBP generation under varying disinfectant concentrations and pH values revealed peak levels at a molar ratio of 25 for LEV and SPN to chloramine, with LEV contributing 11.5% and SPN 23.8% to the relative abundance of DBPs. Remarkably, this research underscores a substantial increase in DBP formation within the molar ratio range of 1:1 to 1:10 compared to 1:10 to 1:25. Furthermore, a pronounced elevation in DBP generation was observed in the pH range of 7 to 8. These findings present critical insights into the impact of the disinfection process on these antibiotics, emphasizing the innovation and significance of this research in assessing associated health risks.

## 1. Introduction

Antibiotics serve not only as crucial agents for disease treatment but also contribute to enhancing livestock productivity through growth promotion [1]. The escalating demand for antibiotics, driven by rapid advancements in public healthcare, animal husbandry, and aquaculture, have become integral to these sectors. However, this surge in antibiotic usage has raised concerns due to the emergence of microbial resistance genes in the environment, posing significant risks to environmental health [2]. Levofloxacin (LEV) and sulfaphenazole (SPN), two synthetic broad-spectrum antibiotics [3], have found widespread application in treating respiratory [4], urinary [5,6], and intestinal infections [7]. Nevertheless, incomplete metabolism has resulted in their frequent detection in surface water [8,9], wastewater [10], and even tap water on a global scale [11]. For instance, LEV has been detected at high levels in domestic wastewater in Shandong Province and in the Lake Taihu basin in China [12,13]. Similarly, the concentrations of SPN and LEV in effluents from large-scale municipal wastewater treatment plants in Guangzhou and Shanghai were 58.33 μg/L and 195.88 ng/L, respectively [11,14]. The inadequacy of conventional water treatment plants in effectively removing antibiotics from water contributes to their ubiquitous detection in the environment [15,16].

It is imperative to recognize the potential transformation of antibiotics, such as LEV and SPN, by disinfectants in the water treatment chain into DBPs with elevated health risks. Chen et al. [17] successfully calculated the genotoxicity of 11 transformation products (TPs) using a 3D-QSAR model, and 10 of the 11 TPs showed higher genotoxicity than the parental LEV. In addition, Ji et al. [18] found that there was a significant increase in the formation of trihalomethanes after the chlorination of tap water containing sulfapyridine in both the water distribution system and the beaker system was performed. This phenomenon poses a noteworthy threat to human health and necessitates a comprehensive understanding of the environmental implications associated with antibiotic use and their subsequent transformation in water treatment processes.

Chlorination disinfection stands as a vital safeguard to ensure clean water resources [19,20,21,22], particularly as a residual disinfectant for controlling microbial regeneration in drinking water distribution system. Chloramine has now become a frequently used disinfectant to reduce the formation of regulated disinfection byproducts (DBPs) such as trihalomethanes (THMs) and haloacetic acids (HAAs) [23]. However, the interactions between chloramine and the dissolved organic compounds in water results in the formation of nitrogen-disinfection byproducts (N-DBPs) [24,25], which are often more toxic than THAs and HAAs [26,27,28,29,30,31]. Previous studies have found that antibiotics can be transformed into N-DBPs by reacting with the disinfectant chloramine; for example, metronidazole could be oxidized by monochloramine disinfectant to form dicholoacetamide (DCAcAm) [32] and oxytetracycline could form the N-nitrosodimethylamine (NDMA) [33], which have been cited as research priorities by the U. S. EPA owing to their greater cytotoxicity and genotoxicity than currently regulated C-DBPs (e.g., THMs) [34]. The intricacies of DBP formation during disinfection are challenging to elucidate due to the influence of various factors such as pH values [35], disinfectant concentration, and inorganic ions [36,37,38]. Previous studies have highlighted the pH-dependent yield of NDMA during chloramine disinfection, emphasizing the significant impact of pH on DBP formation [33]. Zhou et al. [39] observed that the concentration of THMs formed by tetracycline antibiotics during chloramination increased with chloramine concentration. Similar results were reported that higher chloramine concentration led to more natural organic matter being oxidized to dichloroacetonitrile [40]. While the elevation of chloramine dosage has been generally observed to augment DBP formation within a specified range, it is noteworthy that pH exhibits distinct patterns concerning various DBPs [41], such as an initial increase followed by a decrease [42] or a continuous increase within a certain range [43], depending on the specific DBP. This nuanced understanding of pH dynamics is essential for a comprehensive evaluation of the factors governing DBP formation. However, there is a lack of investigation of the formation potential of unidentified DBPs from LEV and SPN under the influence of chloramine concentration and pH. Understanding the mechanisms underlying DBP formation during chloramine disinfection involving LEV and SPN is crucial for deciphering the overall formation patterns of these DBPs. This knowledge contributes to the assessment of potential health risks associated with water disinfection processes involving these commonly encountered antibiotics.

Therefore, the objectives of this study are to: (1) identify the molecular structure of DBPs formed in chloramine disinfection by analyzing the potential reaction sites within the structures of LEV and SPN and by doing so to predict the pathways through which these products are formed; and (2) to conduct kinetic experiments to investigate the influence of various factors, specifically disinfectant concentration and pH, on the formation of DBPs and, consequently, providing guidance for ensuring ecological safety.

## 2. Results and Discussion

### 2.1. Identification of the Chlorination Products of LEV

In this study, levofloxacin, a third-generation fluoroquinolone with a tertiary amine structure similar to trimethylamine, was chosen as the subject for investigating the formation of disinfection byproducts (DBPs) subsequent to its chloramination. As depicted in Table 1, LEV contains reactive sites with three N atoms and two C atoms in its organic structure.

It is anticipated that one or more Cl atoms will be added to the parent compound structure. The reactions between LEV and HOCl, including subsequent chlorine atomic substitution and piperazine ring opening, may occur at different active sites [N(1), N(2), N(3), C(1) and C(2)] [42]. Previous studies have indicated that due to the high electronegativity of N(3), it is more susceptible to HOCl attack, suggesting that the reaction undergone by LEV could occur at this active site and lead to the formation of chlorammonium (LEV^+^Cl) [43,44,45]. This is consistent with the literature by Shah et al. [46,47,48], which suggests that this species could be formed due to the presence of tertiary amine and the addition of ClO^−^. Subsequently, a primary cation could be formed followed by the opening of the 4-methylpiperazine cycle due to internal tension, leading to several pathways with a number of internal eliminations due to the high activity of this cation. Furthermore, nucleophilic attack at the quinolone ring can lead to decarboxylation, and halogenation can subsequently occur at C(1) site to generate several transformation products. To understand the potential sites of attack in the LEV structure and determine the possible DBPs formed during chloramination, several fragmentation pathways along with their product ions were proposed. As shown in Table 2, seven major transformation products were detected during the chlorination disinfection of LEV.

**Figure 1:**
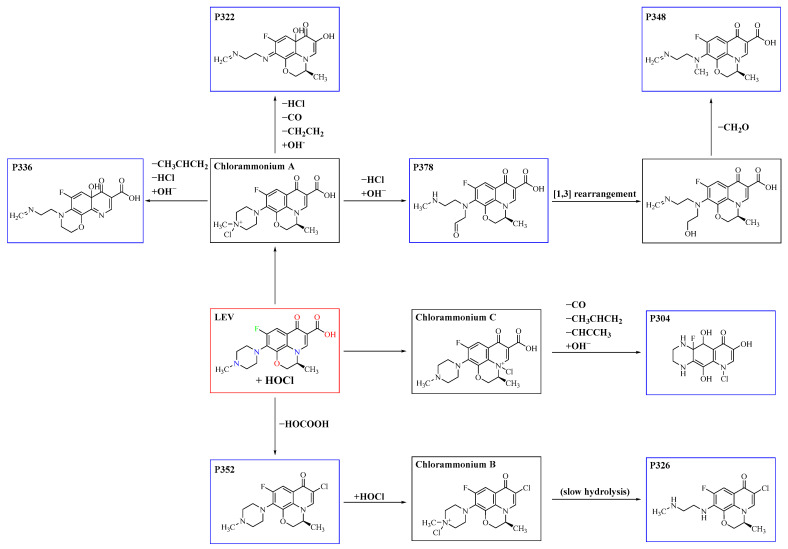
Proposed pathways for the formation of seven DBPs (blue frame) during LEV (red frame) chloramination.

Through structural characterization of the chloramine disinfection products of LEV and incorporating the literature [45], we assumed that chlorammonium intermediates play an important role in the formation pathway of DBPs, as shown in Figure 1. For compounds P322, P336 and P378, it is due to the attack of HOCl at the N(3) reaction site of LEV led to the formation of chlorammonium A. Compound P336 resulted from loss of −HCl (36 amu) and −CH_3_CHCH_2_ (42 amu) followed by the addition of OH^−^, 17 atomic mass unit (aum), while compound P322 was formed through the elimination of −HCl (36 amu), −CO (28 aum) and −CH_2_CH_2_ (28 aum) fragments and subsequent addition of OH^−^ (17 aum). The formation of compound P378 was initiated by the removal of −HCl (36 amu) from the same intermediate, followed by the addition of the −OH (17 aum) group. Subsequently, compound P348 was generated through the elimination of −CH_2_O (30 amu) from compound P378. The loss of the hydroxyl functional group and Cl-substitution led to the formation of compound P352, which could be attributed to a reaction between the parent compound LEV and HOCl at the C(1) site, given its high negative charge. A second attack of HOCl then occurred at the N(3) site of compound P352 to form another chlorammonium product (chlorammonium B), which exhibited a propensity for cleaving the piperazine ring, leading to the formation of compound P326. For the transformation product P304, the addition of a chlorine atom took place on the N(2) site, leading to the formation of chlorammonium C. This, in turn, increased the internal tension of the structure of nitrogen-containing heterocycles, resulting in the successive elimination of −CO (28 amu), −CH_3_CHCH_2_ (42 amu) and −CHCCH_3_ (40 amu) and the addition of an OH^−^ (17 aum) group.

The pivotal role of the piperazine ring’s instability and the susceptibility of its N(3) site to nucleophilic attacks in generating highly active chloramine intermediates during LEV disinfection is underscored. These intermediates are instrumental in delineating the intricate pathways leading to diverse LEV transformation products, classified as Cl-substituted, OH-substituted, and ring-opening derivatives. In-depth insights into these products were attained through the application of LC-MS/MS, providing a detailed understanding of their structural characteristics and fragmentation patterns. This approach enabled a comprehensive elucidation of chloramination reaction pathways, contributing to the identification of specific chemical modifications occurring in the LEV molecule. This study leverages the instability of the piperazine ring and the N(3) site’s susceptibility, marking a significant advancement in comprehending the intricacies of chloramine-induced transformations during LEV disinfection.

**Figure 2:**
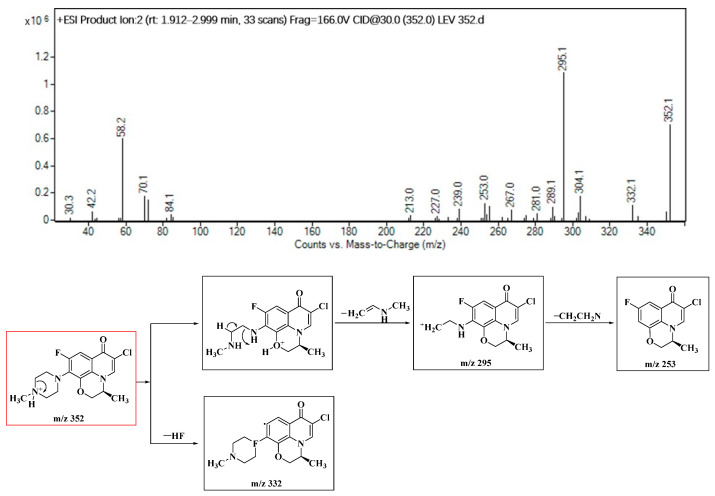
Identification of the molecular structure of P352 (red frame) based on MS/MS.

In this study, seven distinct molecular structures of byproducts resulting from the chloramine disinfection of LEV were identified. The elucidation of the ion fragments was accomplished through secondary mass spectrometry. Taking product P352 as an illustrative example, the secondary mass spectrum is shown in Figure 2. An ion at *m*/*z* 332 originated from the elimination of −HF (20 amu), while the fragment ions at *m*/*z* 295 and *m*/*z* 253 likely derived from the successive elimination of −CH_2_CHNHCH_3_ (57 amu) and −CH_2_CH_2_N (42 amu), respectively. 

The identification and analysis of the molecular structure of the remaining products are illustrated in Figure S1. Upon identifying the structure of P378, fragment ions with *m*/*z* 361, *m*/*z* 334, and *m*/*z* 317 were observed, presumably due to the removal of −OH (17 amu), −CO_2_ (44 amu), and −OH (17 amu), respectively. Similarly, compound P348 displayed fragment ions of *m*/*z* 330 and *m*/*z* 284, resulting from the removal of −F (18 amu) and −CO_2_H_2_ (46 amu). Furthermore, a complex spectrum was observed for compound P336, featuring ions at *m*/*z* 318, *m*/*z* 298, *m*/*z* 279, *m*/*z* 261 and *m*/*z* 235, corresponding to the successive loss of −H_2_O (18 amu), −HF (20 amu), −C_3_H_7_N (57 amu), −F (18 amu) and −CO_2_H_2_ (46 amu). The product ion P326 was identified to produce fragment ions with *m*/*z* 306, 295, 269 and 249, indicating loss of −HF, −CH_3_NH_2_, −C_4_H_9_ and −HF. For P322, fragment ions with *m*/*z* 304, 284, 261 and 235 were observed, linked to the removal of −H_2_O (18 amu), −HF (20 amu), −C_2_H_5_N (43 amu) and −CN (26 amu). In the case of transformation product P304, the initial fragment ion at *m*/*z* 286 resulted from the loss of −H_2_O (18 amu), followed by subsequent eliminations of −CO (28 amu) and −C_2_H_6_N_2_ (58 amu), giving rise to product ions with *m*/*z* 258 and *m*/*z* 228, respectively. The fragment ion at *m*/*z* 228 then underwent successive eliminations of −CO (28 amu), resulting in the final ion with *m*/*z* 200.

### 2.2. Identification of the Chlorination Products of SPN

Sulfaphenazole (SPN) is a sulfanilamide whereby the hydrogen atom of the benzene sulfonamide group is replaced by a 1-phenyl-1*H*-pyrazol-5-yl group. As shown in Table 1, SPN exhibits reactive sites consisting of two N atoms and one C atom. The molecular structures of several disinfection byproducts have been identified by LC-MS/MS. This allows for the comprehensive characterization of the DBPs formed, providing valuable insights into their chemical composition, structural features, and potential health implications. Subsequently, probable pathways have been proposed. Furthermore, owing to the atomic charges associated with aniline-N [N(1)], carbon atom [C(1)], the imino-N [N(2)] within the shared structural framework of the sulfonamide antibiotic, 4-aminobenzenesulfonamide and the O atoms of the −SO_2_ group, all exhibit a negative charge. This insightful observation, as documented by Fu et al. [49], posits that HOCl is capable of targeting these electron-rich regions, thereby fostering electrophilic reactions predominantly at these sites. Consequently, within the context of SPN, it becomes evident that HOCl similarly directs its attack towards these electron-rich reaction sites. The comprehensive details, encompassing retention time, potential molecular formula, ion fragments, and proposed structures for each of the five transformation products, are summarized in Table 3.

It has been documented that sulfonamides are susceptible to various reactions, encompassing Cl-substitution, OH-substitution, S−N bond cleavage, and smile-type rearrangement [49,50]. These reaction categories hold particular significance, suggesting their broad applicability and generalizability to SPN. In the specific instances of Cl-substitution products P383 and P349, disparities in the sites of Cl atom substitution induce the formation of geometric isomers. Such substitutions can manifest at the aniline-N, the imino-N, or the aniline ring structure. Through a detailed analysis of ion fragment structure through secondary mass spectrometry, it is postulated that Cl-substitution initially occurs at the imino-N of the aminobenzene sulfonamide structure, yielding compound P349. Previous studies have shown that electrophilic reagents (e.g., ClO_2_ [51,52] and KMnO_4_ [53,54]) can attack the aniline-N [N(1)] site or the tertiary amine site attached to −SO_2_ group in the structure of sulfonamide antibiotics. In addition, Uetrecht et al. [55] also reported that the substituted products at the ortho-position are generated at very low yields, while several studies reported aniline-N as the active site for sulfonamides [53,56].

The occurrence of further substitution reactions at the aniline-N, leading to the generation of intermediate, is substantiated by the formation of transformation compound P285. Fu et al. [57] identified HOCl’s ability to activate the aniline-N, making substitution on this site the primary driving force for desulphurization, resulting in the lose the –SO_2_ group. Considering SPN’s incorporation of the 4-aminobenzenesulfonamide structure, the leaving group is the sulfonyl group, whose replaceability has been reported [58,59,60]. In addition, due to the presence of lone pair electrons on imidonitrogen, aniline is susceptible to nucleophilic attack to undergo substitution reactions and subsequently experience rearrangement, leading to the loss of the –SO_2_ group and the formation of the benzene-1, 4-diamine cation.

**Figure 3:**
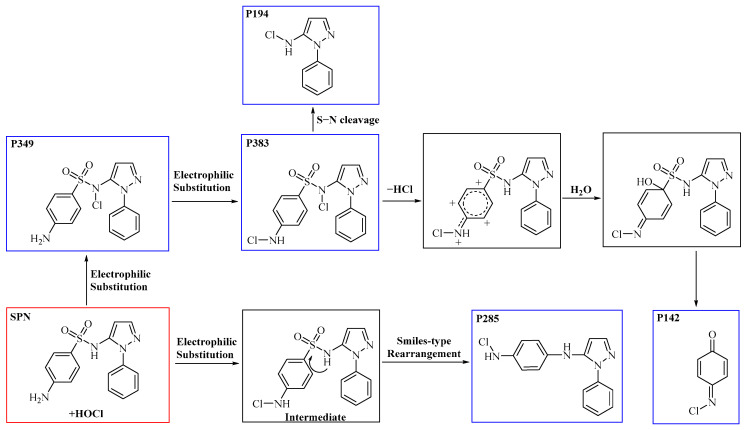
Proposed pathways for the formation of five DBPs (blue frame) during SPN (red frame) chloramination.

Moreover, S–N bond cleavage of P383 gives rise to transformation product P194. The removal of HCl from compound P383, followed by the addition of H_2_O, generates a nitrenium ion intermediate and p-hydroxy adduct, ultimately resulting in the formation of product P142. Notably, P142 is a well-known intermediate formed during the Berthelot reaction for the determination and analysis of ammonia concentration [24]. The proposed reaction pathways for these five transformation products (blue frame) are illustrated in Figure 3.

**Figure 4:**
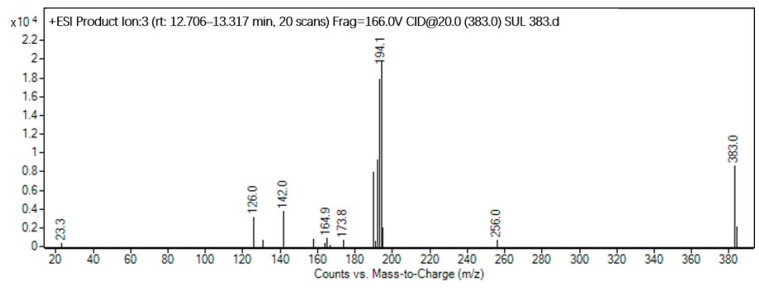

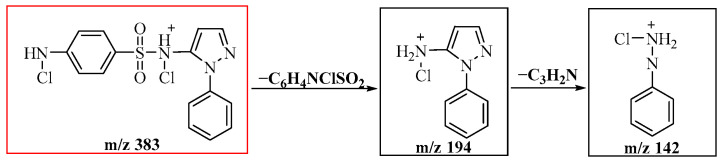
Identification of the molecular structure of P383 (red frame) based on MS/MS.

In this study, we identified five diverse molecular structures of the byproducts formed during the disinfection process of SPN chloramines. Taking product P383 as an example, the secondary mass spectrum is shown in Figure 4. The fragment ions at *m*/*z* 194 and *m*/*z* 142 were observed, specifically, the product ion at *m*/*z* 194 originates from the cleavage of the S−N bond in P383, signifying the loss of −C_6_H_4_NClSO_2_ (189 amu) and −C_3_H_2_N (52 amu), respectively. 

**Figure 5:**
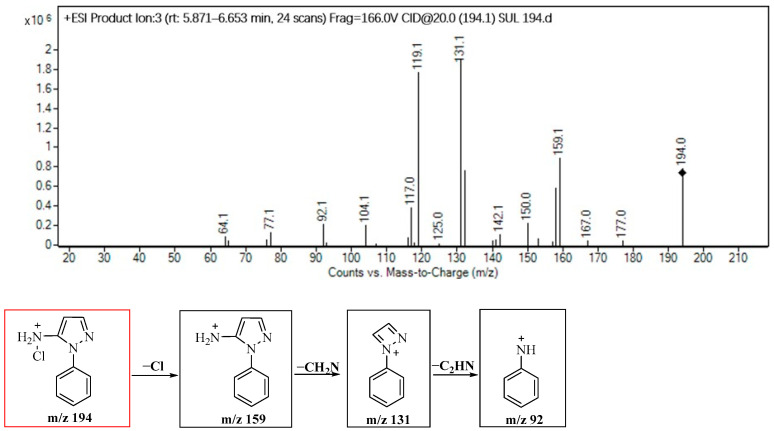
Identification of the molecular structure of P194 (red frame) based on MS/MS.

The elucidation of the molecular structure of the product derived from SPN following chloramine disinfection, as demonstrated in Figure 5, reveals insights into the formation of P194 and the remaining byproducts. Upon subjecting the product ion P194 to applied voltage, subsequent fragmentation was observed, resulting in fragments at *m*/*z* 159, 131 and 92, indicating the sequential loss of −Cl (35 amu), −CH_2_N (28 amu) and −C_2_H_2_N (39 amu), respectively.

**Figure 6:**
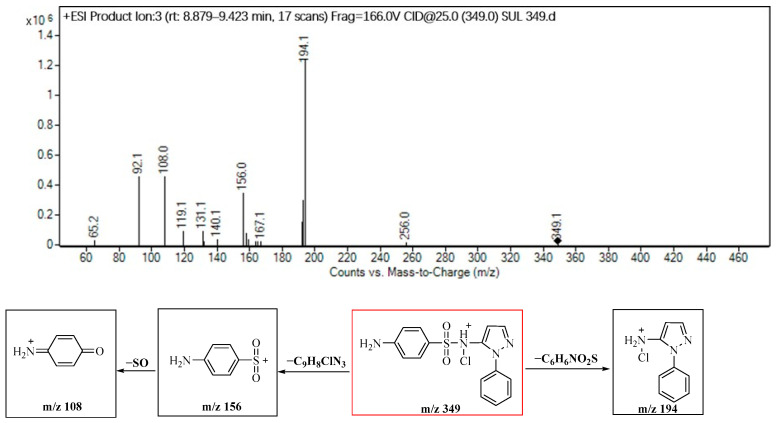
Identification of the molecular structure of P349 (red frame) based on MS/MS.

Another identified product, P349, generates fragment ions at *m*/*z* 194, 156 and 108, indicating the loss of −C_6_H_5_NO_2_S (155 amu), −C_9_H_8_N_3_Cl (193 amu), and −SO (48 amu), respectively. A detailed analysis of the fragment ions reveals that P349 falls within the Cl-substitution product category, localized at the site of the secondary amine nitrogen, as shown in Figure 6.

**Figure 7:**
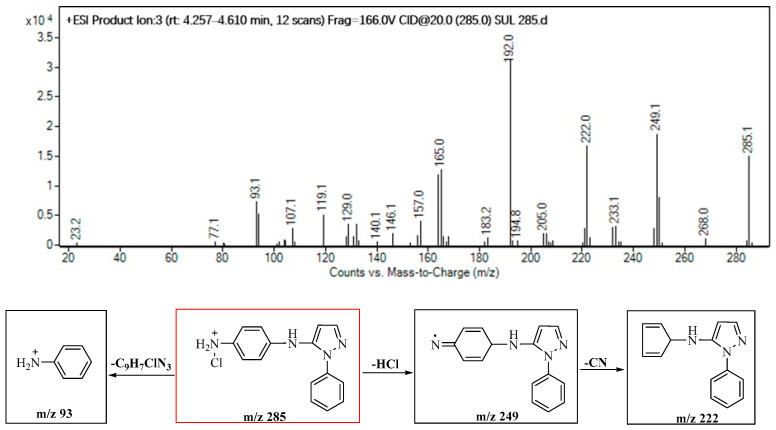
Identification of the molecular structure of P285 (red frame) based on MS/MS.

Product P285, as shown in Figure 7, identified as a Cl-substitution product after desulfurization, fragment ions with *m*/*z* 249, 222 and 93 were observed, representing the loss of −HCl (36 amu), −CN (27 amu) and −C_9_H_7_ClN_3_ (192 amu), respectively. Significantly, our previous work has found that the Cl-substitution reaction primarily initiates the desulfurization reaction. 

**Figure 8:**
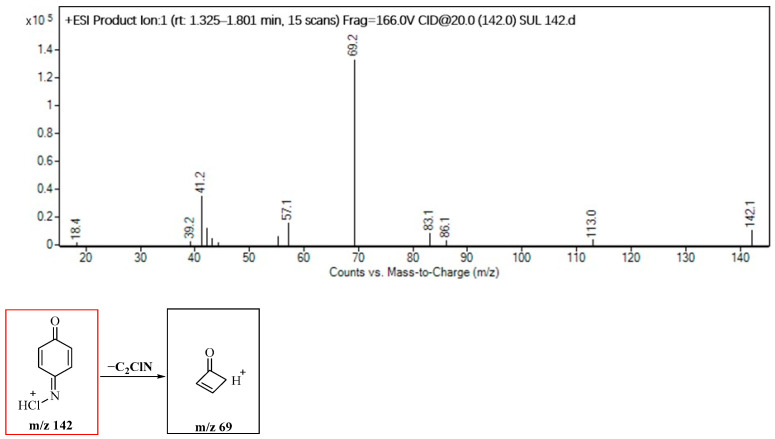
Identification of the molecular structure of P142 (red frame) based on MS/MS.

The MS/MS spectrum of compound P142, also displayed in Figure 8, exhibits a product ion with *m*/*z* 69, attributed to the elimination of −C_2_ClN (73 amu) from compound P142. In this study, observations of Cl-substitution, OH-substitution, S−N bond cleavage, and smile-type rearrangement were obtained. These findings are crucial, providing further evidence that certain reaction types can be generalized for sulfonamides. The data obtained contribute to the development of models capable of predicting the structures of the disinfection byproducts of sulfonamides based on the structures of the parent compounds.

### 2.3. Factors Influencing DBPs Formation

#### 2.3.1. Effect of Chloramine Dosage

**Figure 9:**
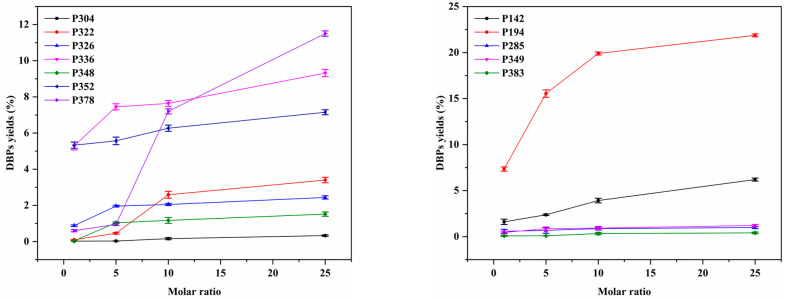
Formation yields of DBPs at different molar ratios.

The concentration of chloramine in disinfection is an important factor to consider when evaluating its impact on water treatment and DBP formation [61]. Figure 9 illustrates the formation of DBPs following the chloramination of LEV and SPN at various disinfectant dosages over a 24 h period. For both antibiotics, the yields of DBPs gradually increased as the chloramine disinfectant concentration varied from 0.05 mmol/L to 1.25 mmol/L, with the maximum yield observed at 1.25 mmol/L. For LEV, the maximum yields for P378, P352, P348, P336, P326, P322, and P304 were recorded at 11.5%, 7.15%, 1.52%, 9.32%, 2.44%, 3.4%, and 0.33%, respectively. Conversely, in the case of SPN, the maximum yields for compounds P383, P349, P285, P194, and P142 were 0.41%, 1.21%, 0.99%, 21.87%, and 6.2%, respectively, at the highest disinfectant dosage of 1.25 mmol/L. The results indicate a significant increase in the formation of DBPs when the molar ratio of the precursor to chloramine disinfectant falls within the range of 1:1~1:10, compared to the range of 1:10~1:25. This observation may be attributed to the fact that, upon reaching the threshold (0.5 mmol/L), an increase in the disinfectant concentration leads to further oxidation of the generated DBPs. In particular, concerning the DBPs formed by LEV, product P378 emerged as a piperazine ring-opening derivative, and its formation (0.54–11.50%) surpassed that of several other DBPs as the concentration of chloramine increased (0.05–1.25 mmol/L), suggesting the superiority of the piperazine ring-opening reaction. The formation of product P336 (9.32%) was lower than that of P378, and the increase in chloramine concentration led to the elevated oxidative capacity of the reaction system, which in turn facilitated the formation of more hydroxylated products. In addition, the formation of product P352 (Cl-substituted product) showed a similar trend to that of P378, registering a formation rate of 7.15%, significantly higher than that of other DBPs. We speculated the other DBPs might represent terminal products, necessitating the transition of intermediates. Despite the escalating chloramine concentration promoting the oxidation of more precursors, it led to the formation of less pronounced terminal DBPs. Shifting focus to the DBPs formed by SPN, a notable observation is the markedly higher formation of P194 (S−N bond cleavage product) at 21.87%, compared to the other four DBPs. This underscores the susceptibility of the tertiary amine functional group to oxidative cleavage during the chloramination process of SPN. The concentration of P142 (oxidized product) (6.2%) also exhibited an increasing trend with the elevation of the chloramine concentration. However, while the concentrations of several other DBPs increased alongside chloramine concentration, their overall concentration remained consistently low. This suggests that the structure of the reaction intermediates is prone to oxidation by chloramine, ultimately resulting in the formation of the S−N bond cleavage product and the oxidation product.

#### 2.3.2. Effect of pH Values

**Figure 10:**
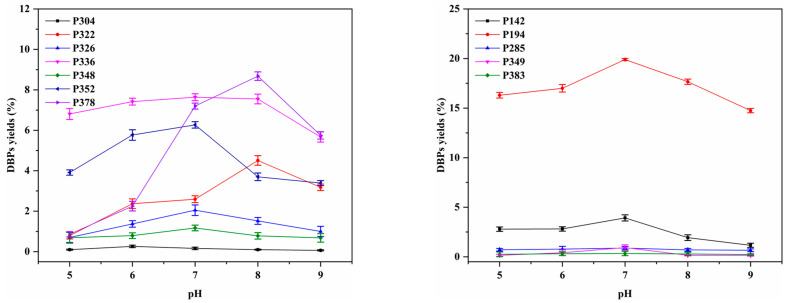
Effect of pH on DBPs formation from 24 h chloramination of LEV and SPN.

Previously, Gros et al. [62] and Zhou et al. [39] extensively investigated the impacts of solution pH on monochloramine auto-decomposition and hydrolysis reactions. Consequently, alterations in pH would induce variations in the yield of chloramine disinfection products from LEV and SPN, as illustrated in Figure 10.

The formation of seven DBPs formed by LEVs during chloramine disinfection showed various trends in the pH range of 5 to 9. The formation of P322, P326, P336, P348, P352 and P378 showed an upward trend when the pH ranged from 5 to 7. The formation of all seven DBPs showed a downward trend when the pH ranged from 8 to 9. In addition, P326, P336, P348, P352 and P304 showed a downward trend already from pH = 7. However, for P378 (open-ring oxidation product), the yield at pH = 8 exhibited a notable increase, reaching 8.68%, surpassing the yields at other pH values. This observation implies a distinct advantage in the formation process under pH = 8 conditions. Additionally, P322 (4.51%), P336 (7.64%), a piperazine ring-opening product), and P352 (6.27%, Cl-substitution product) also displayed significantly higher yields in the pH range of 7 to 8 compared to other products such as P304 (0.26%), P348 (1.17%), and P326 (2.05%). 

In the case of SPN, compound P194 (S−N bond cleavage product) emerged as the primary DBP formed during the 24 h chloramination process at pH 7, achieving a maximum yield of 19.9%. The yields of the remaining DBPs were comparatively lower, with maximum yields of 3.92%, 0.88%, 0.93%, and 0.33% for compounds P142, P285, P349, and P383, respectively. The variations in product yields could be attributed to differences in the dissociated forms of chloramine hydrolysis and precursors. At pH = 7 to 8, chloramine hydrolysis yielded the formation of HOCl with enhanced oxidative properties, indicating the generation of HOCl under neutral pH conditions during the hydrolysis of NH_2_Cl. The low DBP yields at pH = 5 can be explained by the decomposition of NH_2_Cl into NHCl_2_ through acid-catalyzed NH_2_Cl disproportionation. Conversely, the low yields at alkaline pH (pH 9) could be attributed to base-catalyzed hydrolysis, which may inhibit the formation of HOCl. 

These results underscore the significant influence on the formation potential of DBPs during the chloramination of LEV and SPN. Based on these findings, it can be seen that DBPs were produced at the lowest concentrations by chloramination when the exposure time was 24 h, pH = 9, and the molar ratio was less than 1:10.

## 3. Materials and Methods

### 3.1. Chemical Reagents and Materials

All the chemical solutions used in this research were prepared with ultra-pure water from the Milli-Q Progard water purification system (Ningbo Dansibuodun Environmental Protection Technology Co., Ltd., Ningbo, China) and analytical reagent (AR) grade chemicals. Sodium hypochlorite (NaOCl, available chlorine 4.4%) and ascorbic acid were obtained from Shanghai Aladdin Biochemical Technology Co., Ltd. (Shanghai, China) and Shanghai Zhanyun Chemical Co., Ltd. (Shanghai, China), respectively. The levofloxacin and sulfaphenazole were purchased from Meryer Biochemical Technology Co., Ltd. (Shanghai, China) and Shanghai Macklin Biochemical Co., Ltd. (Shanghai, China), respectively. Ammonium chloride (NH_4_Cl, 99.5%) was purchased from Sinopharm Chemical Reagent Co., Ltd. (Shanghai, China). The molecule structures of the LEV and SPN are listed in Table 1.

### 3.2. Chloramine Disinfection

In this study, chloramine disinfection experiments were conducted in 100 mL amber glass bottles in the dark, and the experiment was carried out in a constant temperature incubator, maintaining a controlled temperature of 25 ± 0.5 °C. To stabilize the pH value of the reaction system, 10 mmol/L phosphate buffer was added. In order to clearly identify the molecular structures of the yielded DBPs, concentrations of 0.05 mmol/L for both LEV and SPN were employed in this study.

The molar ratio of NH_2_Cl to the precursors varied from 1:1, 5:1, 10:1 to 25:1, and the pH levels ranged from 5, 6, 7, 8 to 9. At pre-selected intervals, residual free chlorine concentrations were measured using the N,N-diethyl-p-phenylenediamine (DPD) colorimetric method [63], as previously described. Subsequently, the disinfectant residual was quenched with ascorbic acid to halt the reaction.

### 3.3. Molecular Characterization of DBPs

In this study, the molecular structure of the transformation products obtained from LEV and SPN during chloramine disinfection process was characterized. The samples after the disinfection process were pretreated by solid phase extraction (SPE). Subsequently, the extraction solutions were evaporated under a gentle stream of nitrogen to near dryness, reducing the final volume of the extract to 1 mL. The concentration of LEV and SPN were analyzed using an Agilent 1260 High-Performance Liquid Chromatography (HPLC) system (Agilent Technologies, Palo Alto, CA, USA). The analysis was conducted on an EC-C18 column (3.0 × 100 mm) with a flow rate of 1.0 mL/min. The column temperature was maintained at 30 °C, and a 10 μL sample size was injected. The instrument was equipped with a fluorescence detector (FLD), and the Detection wavelengths for LEV was set at 286 nm while that of SPN was set at 265 nm. The mobile phase was a mixture of acetonitrile (ACN) and a 0.1% formic acid aqueous solution. 

Then, the structure and concentration of DBPs were analyzed by liquid chromatography-mass spectrometry (LC-MS/MS) (Agilent 1290 Infinity II). Mass spectrometry analyses were conducted in electrospray positive ionization (ESI+) mode with a dry gas temperature of 200 °C and a dry gas flow rate of 11 mL/min. The mass/charge ratio was scanned over the range of *m*/*z* 50 to 1000. A collision-induced voltage of 166 V was applied, and the Capillary voltage was set at 4500 V. Chromatographic separation was achieved using an EC-C18 column (Poroshell 120, EC-C18, 2.1 × 100 mm, 2.7 μm, Agilent Technologies, Santa Clara, CA, USA). Sample enrichment was performed using a solid phase extraction column (Bond Elut-C18, 50 mg, 3 mL, 50/PK, Agilent Technologies, USA).

For LEV: the following gradient elution program was employed: 0 to 3 min, a linear increase from 5% B to 20% B; 3 to 15 min, 40% B; 15 to 20 min, a decrease from 40% B to 5% B, followed by equilibration at 5% B. For SPN: 0 to 3 min, a linear increase from 5% B to 20% B; 3 to 5 min, 40% B; 5 to 7 min, an increase from 40% B to 60% B; 7 to 15 min, a decrease from 60% B to 5% B.

## 4. Conclusions

The molecular structures of chloramine disinfection products originating from levofloxacin and sulfamethoxazole were successfully elucidated in this study, revealing seven chloramination products for LEV (categorized as Cl-substituted, OH-substituted, and piperazine ring-opening/oxidized) and five chlorination products for SPN (categorized as Cl-substituted, S−N bond cleavage, and oxidized). The formation pathways of disinfection byproducts (DBPs) were also speculated upon. During the chloramination process of LEV, the N(3) site of the piperazine ring exhibited high reactivity, serving as the primary reaction site for chloramine disinfection. This site favored piperazine ring-opening/oxidation, leading to the formation of chloramine intermediates, subsequently undergoing Cl-substitution and hydroxyl substitution. Similarly, in the chloramine disinfection of SPN, the Cl-substitution reaction drove the desulfurization rearrangement reaction, with preferential S−N bond cleavage occurring without the formation of intermediates.

The generation of DBPs was collectively influenced by chloramine concentration and pH values. Peak DBP generation was observed at a chloramine concentration of 1.25 mmol/L, with the highest incremental generation occurring within the chloramine concentration range of 0.05~0.5 mM, followed by a more stable incremental generation. Additionally, DBP generation was significantly elevated in the pH range of 7~8, attributed to enhanced chloramine hydrolysis forming HOCl, thereby augmenting oxidative capacity. The dissociation ability of precursors and the formation of more reactive species played a crucial role in reacting with disinfectants within this pH range. 

In conclusion, this study on chloramine disinfection of antibiotics, specifically LEV and SPN, holds significant implications for advancing water treatment processes. Key applications include enhancing water quality monitoring for early detection and control of antibiotic contaminants, guiding water treatment optimization to effectively remove antibiotics and reduce DBPs, providing crucial data for policymakers to develop targeted regulations for a safe public water supply, inspiring the development of innovative, sustainable water treatment technologies to address antibiotic impacts, and assisting in designing strategies to mitigate potential health risks associated with stable disinfection byproducts, thereby contributing to public health assurance.

## Figures and Tables

**Table 1:** The molecular structures of antibiotic precursors.

Compound	Molecular Structure	Formula	Molecular Weight	Reactive Sites
Levofloxacin	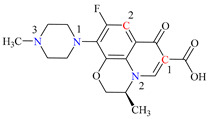	C_18_H_20_FN_3_O_4_	361.37	Piperazine ring-N[N(1),N(2)] Heterocyclic-N[N(1)], C[C(1)]
Sulfaphenazole	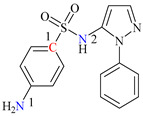	C_15_H_14_N_4_O_2_S	314.36	Aniline-N[N(1)] Benzene ring-C[C(1)]Imino-N[N(2)]

**Table 2:** Detection of products information of LEV in chloramination process by LC-MS/MS.

Compound	Retention Time (min)	Molecular [M + H^+^]	Product Ion	Proposed Structure
LEV	4.8	362	344, 318, 261	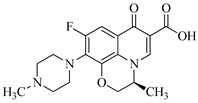
P378	7.2	378	361, 334, 317	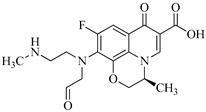
P352	3.9	352	332, 295, 253	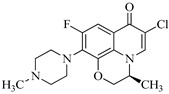
P348	5.1	348	330, 304, 284, 261	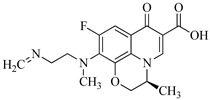
P336	5.0	336	318, 298, 279, 261, 235	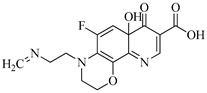
P326	4.0	326	306, 295, 269, 249	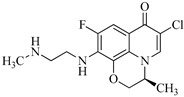
P322	4.9	322	304, 284, 261, 235	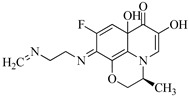
P304	2.4	304	286, 258, 228, 200	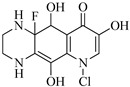

**Table 3:** Detection of products information of SPN in chloramination process by LC-MS/MS.

Compound	Retention Time (min)	Molecular [M + H^+^]	Product Ion	Proposed Structure
SPN	7.9	315	222, 158, 131, 92	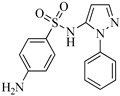
P383	10.1	383	194, 142	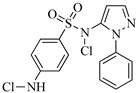
P349	4.9	349	194, 156, 108	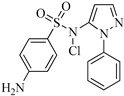
P285	7.5	285	249, 222, 93	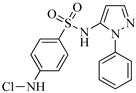
P194	8.6	194	159, 131, 92	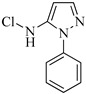
P142	2.7	142	69	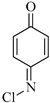

## Data Availability

Data are contained within the article and Supplementary Materials.

## Supplementary Materials

The following supporting information can be downloaded at: https://www.mdpi.com/article/10.3390/molecules29020396/s1, Figure S1: Secondary mass spectrometry of the by-products of chloramine disinfection of LEV.
