# Supplementary material for: Chloramine Disinfection of Levofloxacin and Sulfaphenazole: Unraveling Novel Disinfection Byproducts and Elucidating Formation Mechanisms for an Enhanced Understanding of Water Treatment
Source: Molecules. 2024 Jan 13;29(2):396. doi: 10.3390/molecules29020396 (PMC10820186; doi:10.3390/molecules29020396)
Supplement: Supplementary file 1 [file molecules-29-00396-s001.zip › molecules-2783030-supplementary.pdf]

Supporting Information

Chloramine Disinfection of Levofloxacin and Sulfaphenazole: Unraveling Novel Disinfection Byproducts and Elucidating Formation Mechanisms for an Enhanced Understanding of Water Treatment

Zhenkun Sun, Zhenyi Chen, Marie Celine Chung Lan Mow, Xiaowen Liao, Xiaoxuan Wei *, Guangcai Ma, Xueyu Wang and Haiying Yu *

College of Geography and Environmental Sciences, Zhejiang Normal University, Yingbin Avenue 688, Jinhua 321004, China

* Correspondence: xxwei@zjnu.edu.cn (X.W.); yhy@zjnu.cn (H.Y.); Tel.: +86-0579-82282485 (H.Y.)

Figure

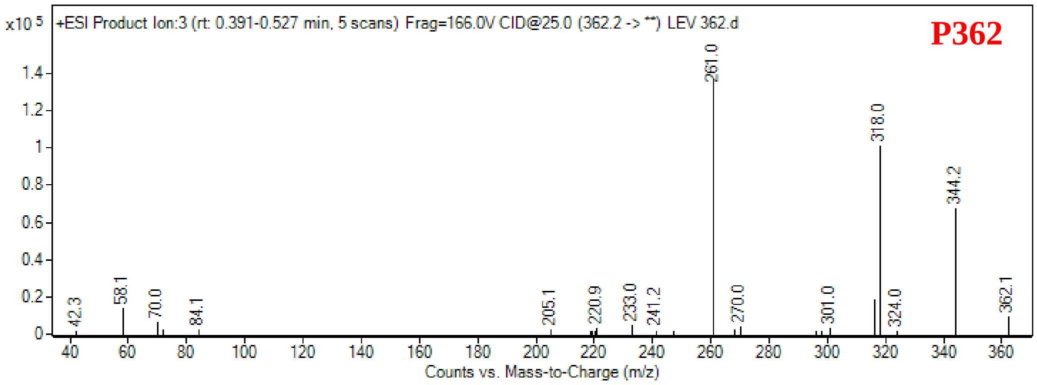

P362
+ESI Product Ion:3 (rt: 0.391-0.527 min, 5 scans) Frag=166.0V CID@25.0 (362.2 -> **) LEV 362.d
x10 5
Counts vs. Mass-to-Charge (m/z)
42.3
58.1
70.0
84.1
205.1
220.9
233.0
241.2
261.0
270.0
301.0
318.0
324.0
344.2
362.1

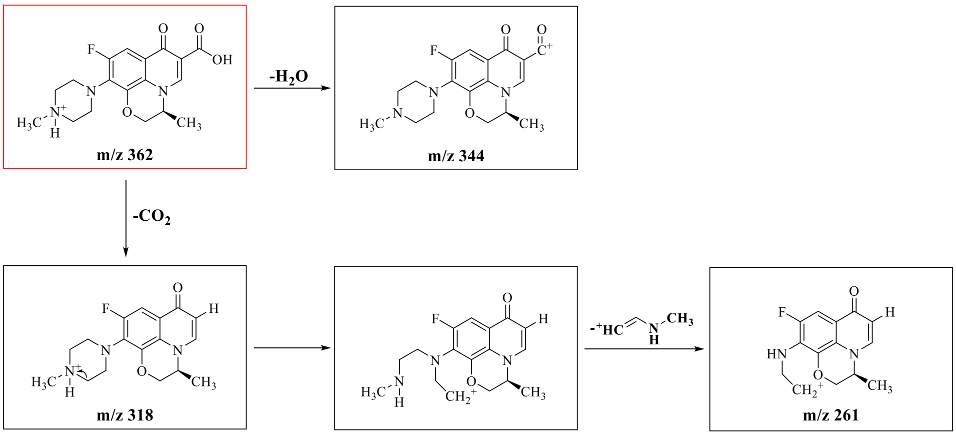

m/z 362
-H2O
m/z 344
-CO2
m/z 318
m/z 261

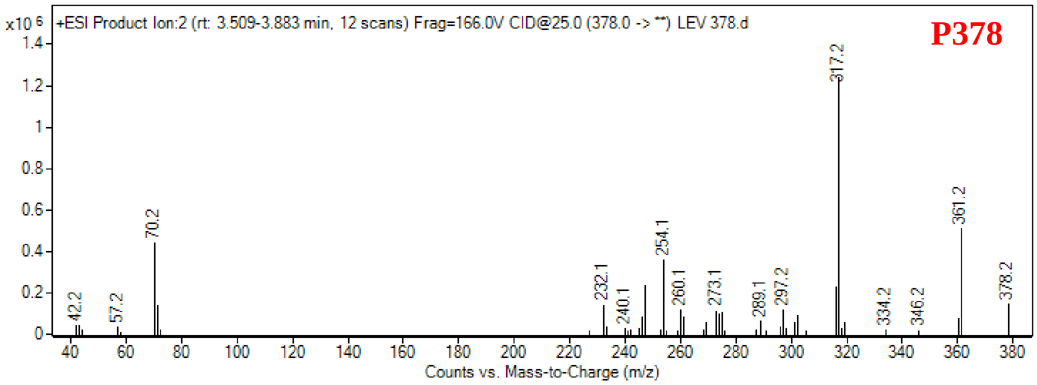

P378
+ESI Product Ion:2 (rt: 3.509-3.883 min, 12 scans) Frag=166.0V CID@25.0 (378.0 -> **) LEV 378.d
x10 6
Counts vs. Mass-to-Charge (m/z)
42.2
57.2
70.2
232.1
240.1
254.1
260.1
273.1
289.1
297.2
317.2
334.2
346.2
361.2
378.2

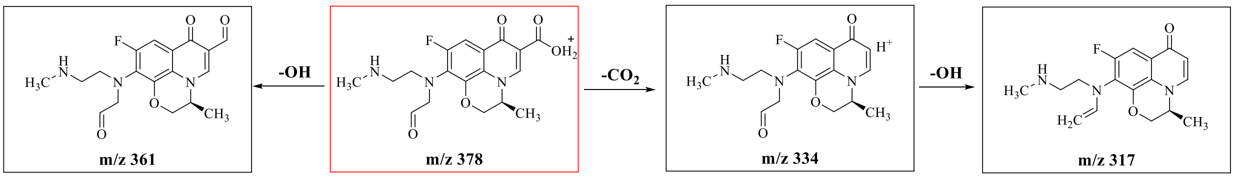

m/z 361
-OH
m/z 378
-CO2
m/z 334
-OH
m/z 317

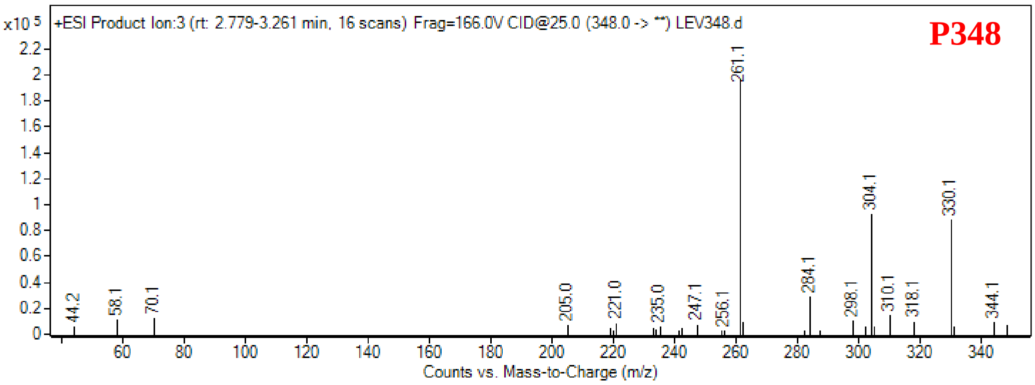

P348
+ESI Product Ion:3 (rt: 2.779-3.261 min, 16 scans) Frag=166.0V CID@25.0 (348.0 -> **) LEV348.d
x10 5
Counts vs. Mass-to-Charge (m/z)
44.2
58.1
70.1
205.0
221.0
235.0
247.1
256.1
261.1
284.1
298.1
304.1
310.1
318.1
330.1
344.1

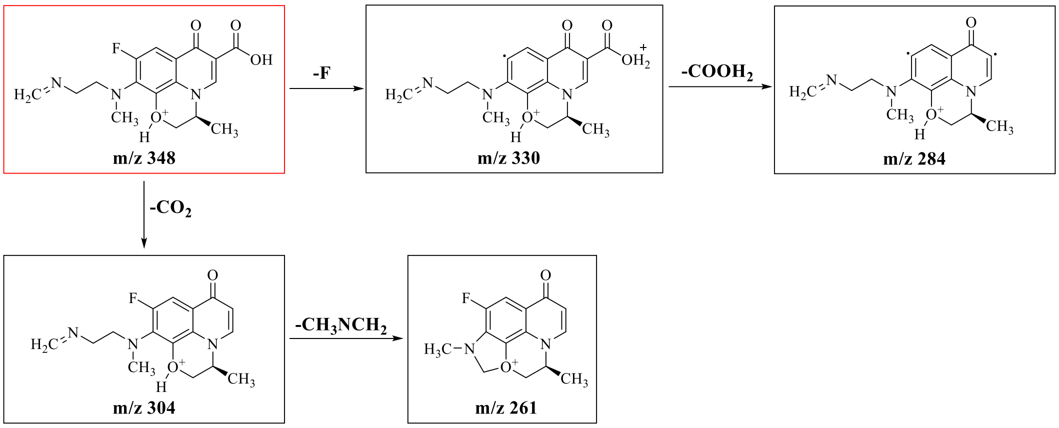

m/z 348
-F
m/z 330
-COOH2
m/z 284
-CO2
m/z 304
-CH3NCH2
m/z 261

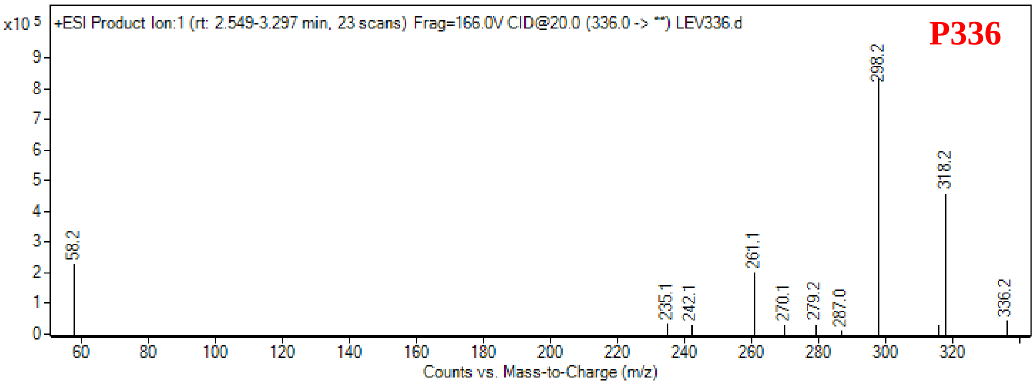

P336
+ESI Product Ion:1 (rt: 2.549-3.297 min, 23 scans) Frag=166.0V CID@20.0 (336.0 -> **) LEV336.d
x10 5
Counts vs. Mass-to-Charge (m/z)
58.2
235.1
242.1
261.1
270.1
279.2
287.0
298.2
318.2
336.2

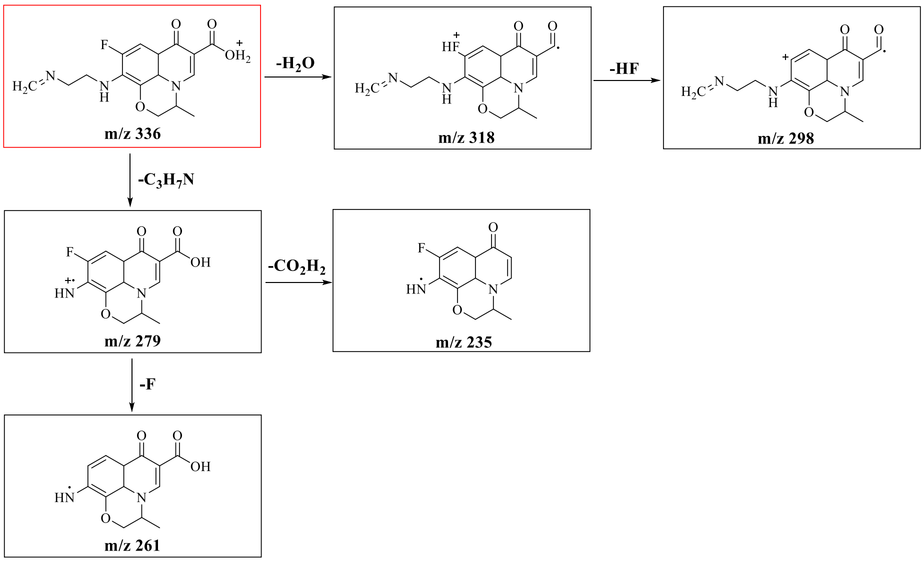

m/z 336
-H2O
m/z 318
-HF
m/z 298
-C3H7N
m/z 279
-CO2H2
m/z 235
-F
m/z 261

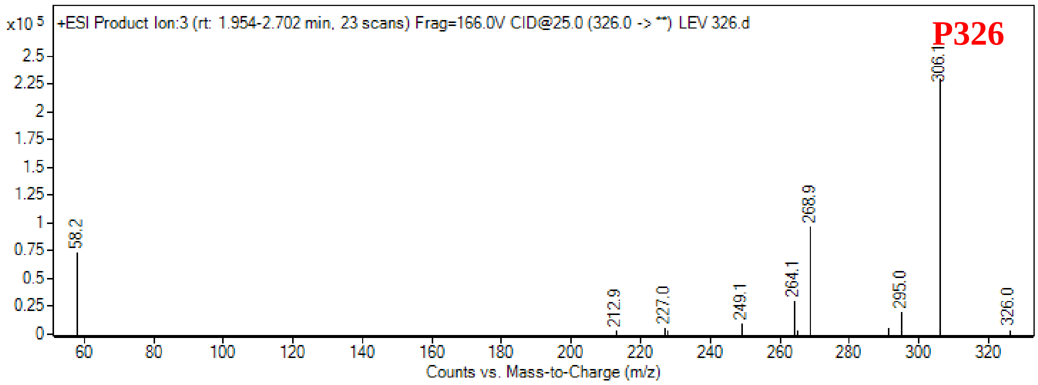
P326
+ESI Product Ion:3 (rt: 1.954-2.702 min, 23 scans) Frag=166.0V CID@25.0 (326.0 -> **) LEV 326.d
x10 5
Counts vs. Mass-to-Charge (m/z)
58.2
212.9
227.0
249.1
264.1
268.9
295.0
306.1
326.0

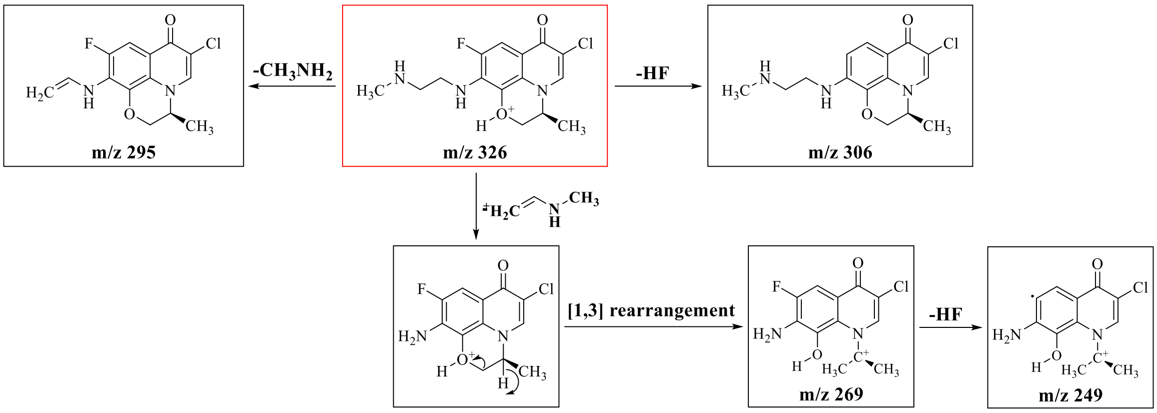
-CH3NH2
-HF
m/z 295
m/z 326
m/z 306
[1,3] rearrangement
-HF
m/z 269
m/z 249

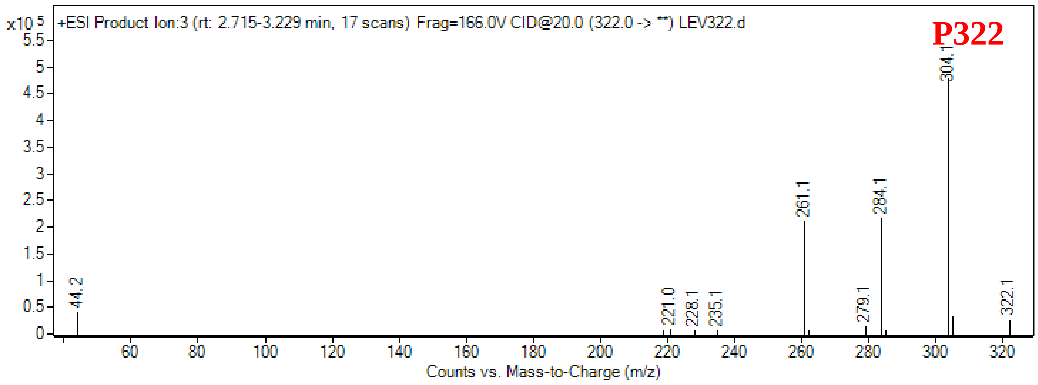
P322
+ESI Product Ion:3 (rt: 2.715-3.229 min, 17 scans) Frag=166.0V CID@20.0 (322.0 -> **) LEV322.d
x10 5
Counts vs. Mass-to-Charge (m/z)
44.2
221.0
228.1
235.1
261.1
279.1
284.1
304.1
322.1

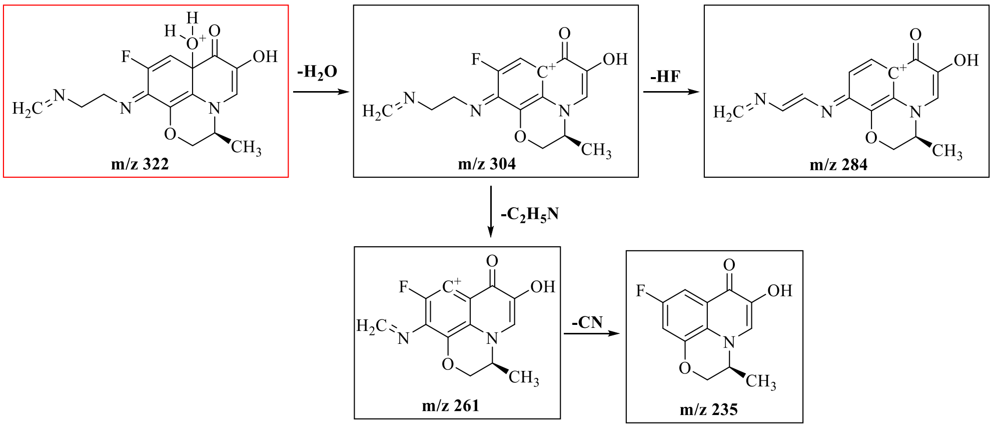
-H2O
-HF
m/z 322
m/z 304
m/z 284
-C2H5N
-CN
m/z 261
m/z 235

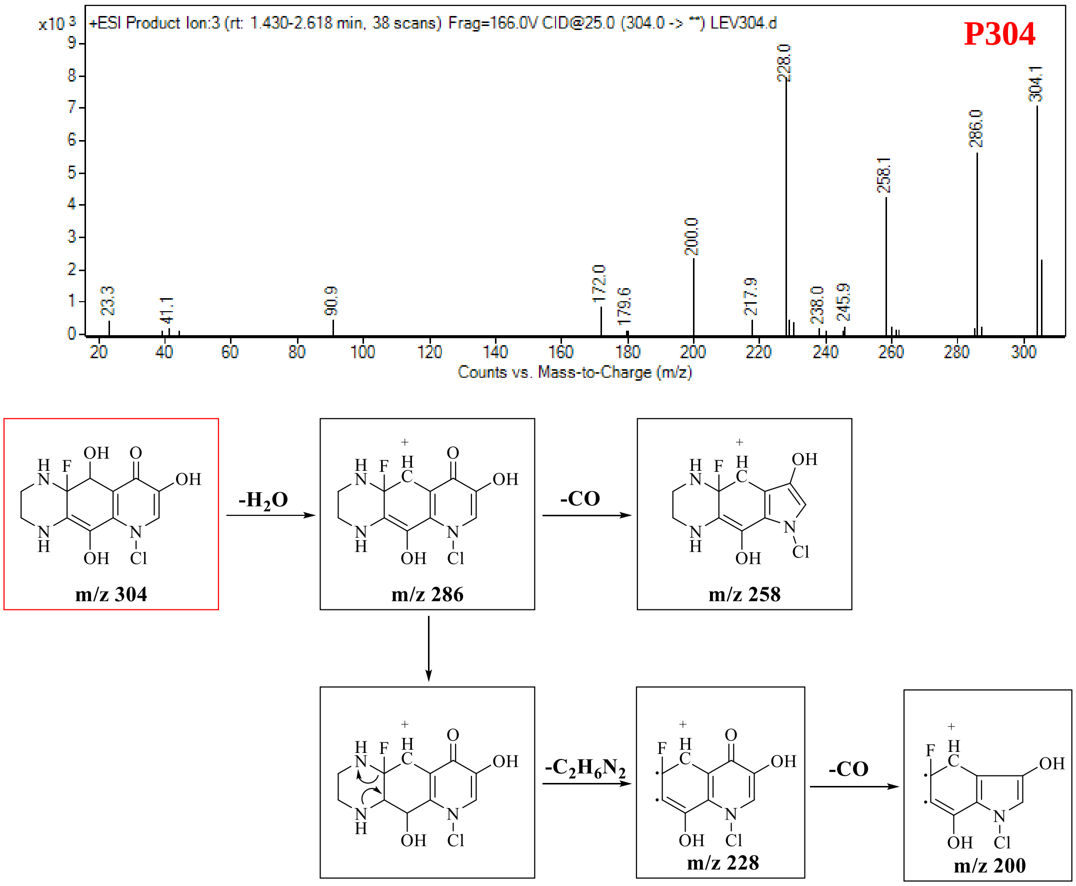

Figure S1. Secondary mass spectrometry of the by-products of chloramine disinfection of LEV.
